# Exploring the Functional Impact of Individual *DDX41* Variants With a Fast and Robust Cell‐Based Method

**DOI:** 10.1155/humu/3758915

**Published:** 2026-06-25

**Authors:** Nikolaj Juul Nitschke, Marwa Almosailleakh, Issa Ismail Issa, Clara Amanda Skov, Casper Carstens Lund, Jakob Werner Hansen, Jakob Schmidt Jespersen, Klas Raaschou-Jensen, Claudia Schöllkopf, Marianne Tang Severinsen, Bo Porse, Joachim Weischenfeld, Anne Stidsholt Roug, Mette Klarskov Andersen, Morten Frödin, Kirsten Grønbæk

**Affiliations:** ^1^ Department of Hematology, Rigshospitalet, Copenhagen University Hospital, Copenhagen, Denmark, gentoftehospital.dk; ^2^ Biotech Research and Innovation Centre (BRIC), Faculty of Health and Medical Sciences, University of Copenhagen, Copenhagen, Denmark, ku.dk; ^3^ Finsen Laboratory, Copenhagen University Hospital-Rigshospitalet, Copenhagen, Denmark; ^4^ Department of Hematology, Odense University Hospital, Odense, Denmark, ouh.dk; ^5^ Department of Hematology, Clinical Cancer Research Center, Aalborg University Hospital, Aalborg, Denmark, aalborguh.rn.dk; ^6^ Department of Clinical Medicine, Aalborg University, Aalborg, Denmark, aau.dk; ^7^ Department of Clinical Medicine, Faculty of Health and Medical Sciences, University of Copenhagen, Copenhagen, Denmark, ku.dk; ^8^ Department of Hematology, Aarhus University Hospital, Aarhus, Denmark, auh.dk; ^9^ Department of Molecular Medicine, Aarhus University Hospital, Aarhus, Denmark, auh.dk; ^10^ Department of Clinical Genetics, Rigshospitalet, Copenhagen University Hospital, Copenhagen, Denmark, gentoftehospital.dk

**Keywords:** AML, DDX41, functional genetics, germline predisposition to myeloid neoplasms, MDS, variant interpretation

## Abstract

Germline *DDX41* variants are found in up to 4% of patients with acute myeloid leukemia (AML) and myelodysplastic neoplasms (MDSs), with a higher prevalence among males and a late onset. In this study, we analyzed 647 Danish patients with suspected myeloid neoplasms to assess the frequency of germline *DDX41* variants and the functional impact of variants of uncertain significance (VUSs) using a cell‐based assay, CRISPR‐Select. We identified 16 *DDX41* variants in 30 patients, 14 of whom were confirmed or predicted as germline variants. Eight germline variants were classified as likely pathogenic/pathogenic (LP/P), and three as VUSs. We generated a monoallelic *DDX41* K562 cell line and used it for CRISPR‐Select assessment of variant effects on proliferation and survival. Pathogenic variants impaired proliferation, while benign variants did not. Missense variants had a more subtle effect than truncating ones, suggesting hypomorphic phenotypes. One *DDX41* VUS had a significant negative effect on proliferation, suggesting it to be pathogenic, while the other two had no effect, suggesting them to be benign. These findings confirm a 2.4% frequency of LP/P DDX41 germline variants in Danish patients and demonstrate the value of CRISPR‐Select in distinguishing pathogenic from benign variants.

## 1. Introduction

In 2015, Polprasert et al. identified *DDX41* germline variants in patients with myeloid neoplasms (MNs) [1]. Since then, several studies have examined the frequency of germline *DDX41* variants and found that up to 4% of all patients with acute myeloid leukemia (AML) or myelodysplastic neoplasms (MDSs) harbor pathogenic variants. In 2022, Makishima et al. examined 9082 patients with MN and identified germline likely pathogenic *DDX41* variants (likely pathogenic/pathogenic [LP/P]) in 293 patients (3.2%) [2]. Kovilakam et al. examined the carrier rate of *DDX41* variants in the UK Biobank and strikingly found that 1/129 and 1/430 carried a nonsynonymous variant (LP/P) [3]. This makes germline *DDX41* variants the most frequent known germline alteration predisposing to the development of MNs. Germline *DDX41* variant–associated MNs (MN‐*DDX41^g^*) have a late age of onset compared to other cancer predisposition syndromes, with a median age of approximately 68 years and a higher prevalence among males, who account for up to 80% of patients [2]. Compared to MDS and AML patients without *DDX41* germline variants (MN‐*DDX41* ^WT^), MN‐*DDX41^g^* patients have a better overall prognosis but a higher risk of AML transformation from MDS [2, 4]. Furthermore, recent studies suggest high response rates to standard treatments such as azacitidine [2, 5], venetoclax [6–8], and melphalan compared to MN‐*DDX41* ^WT^.

Pathogenic germline variants found in *DDX41* are predicted to be loss‐of‐function missense variants, suggesting a role for DDX41 in tumor suppression. *DDX41* encodes a DEAD‐box helicase containing the Asp‐Glu‐Ala‐Asp (DEAD) amino acid sequence [9]. Helicases are a family of enzymes that can unwind DNA and RNA, remove DNA/RNA‐bound proteins, and remodel chromatin [10]. DDX41 is involved in several cellular processes, such as R‐loop resolution [11, 12], splicing [13], ribosome biogenesis [14], and immune surveillance [15]. However, the role of DDX41 in the development of MDS and AML remains unknown [10], and functional models have so far only focused on the knockout of *DDX41* and the knock‐in of the somatic hotspot mutation R525H in mice. Moving forward in understanding and treating *DDX41*‐related disease, a way to correctly classify *DDX41* variants is urgently needed. We, here, report the frequency of germline *DDX41* variants in a Danish cohort and, furthermore, develop the first human cell system and functional genetic assay that allows precise quantitation of the functional effects of individual DDX41 variants in an endogenous genetic context.

## 2. Methods

The description of the patient cohort, the next‐generation sequencing panel, the DNA purification, polymerase chain reaction (PCR), Sanger sequencing, and cell culture has previously been published by Nitschke et al. in *Human Mutation* [16]. Furthermore, CRISPR‐Cas9 editing and CRISPR‐Select methods have also previously been described by Nitschke et al. in *Human Mutation* and by Niu et al. in *Nature Genetics* [16, 17].

### 2.1. Patient Cohort

This study is part of the Danish research program: the Program for Translational Hematology (PTH), approved by the Danish National Ethics Committee (Doc. No.: 1083691671347). Patients referred with suspicion of MN or with suspicion of relapse/progression of existing MN, excluding myeloproliferative neoplasms (MPNs), were included in PTH between 2018 and 2023. On the first visit to a Danish hematological department, informed written consent was obtained, and a skin biopsy, peripheral blood samples, and a bone marrow aspirate were drawn. Skin biopsies were taken from the area above the posterior superior iliac spine and frozen dry at −80°C. We excluded patients from the study if the clinical work‐up provided sound evidence of a non‐MN as the cause of the patient′s symptoms (e.g., acute lymphoblastic leukemia). The diagnostic classifications were based on the clinical findings and clinical NGS data. Therefore, in the current paper, patients with idiopathic cytopenia of unknown significance (ICUS) may harbor somatic variants—including acquired *DDX41* mutations—detected by the broader PTH NGS panel but remain classified as ICUS because the clinical NGS panels were more limited in scope and did not include *DDX41*.

### 2.2. Next‐Generation Sequencing Panel

Six hundred and forty‐seven patients were sequenced as part of the PTH research program using DNA extracted from live frozen mononuclear cells separated with LeucoSep (Greiner Bio‐One) from either peripheral blood or bone marrow aspirates drawn at inclusion.

First, whole‐genome NGS libraries were prepared from 100 ng genomic DNA with the Twist Library Preparation EF Kit 1.0 (Twist BioScience, San Francisco, California, United States) using Universal Adapter Ligation and seven cycles of PCR for unique dual indexing and amplification. Libraries were pooled by equal mass in multiplex (8‐ to 17‐plex) for hybridization capture. Total library DNA in each hybridization reaction was 3500 or 4000 ng. Target enrichment was performed with a custom Twist capture probe panel (Twist BioScience) and the Twist Hybridization and Wash Kit Version 1 using Twist Universal Blockers (Twist BioScience). Hybridization duration was 16–18 h.

The custom Twist panel covers 146 genes related to MNs, including all genes known to be recurrently mutated in clonal hematopoiesis, MDS, and AML based on targets provided by the International Working Group for Prognosis in MDS (IWG‐PM), as listed in Table S1. The target regions of these genes were (all or selected) coding exons, promoter regions (of TERT and TERC only), and the genomic sequence of noncoding genes (microRNAs). For the purposes of this study, we focused our analysis on a subset of 39 genes in which all identified variants were manually curated and reported (Table S1).

The panel capture probe design was performed by Twist BioScience using a proprietary algorithm with 1× tiling of the target regions, resulting in 6373 probes synthesized as 120‐bp dsDNA baits. Capture pools with enriched libraries were sequenced on an Illumina NextSeq 500 instrument (Illumina, San Diego, California, United States) with a PE76 read configuration or an Illumina NovaSeq 6000 (Illumina) with a PE157 read configuration to obtain an average target coverage of > 500× (unique reads) per sample.

We used BWA [18] to align reads to GRCh38, Picard tools [19] to sort the reads and mark duplicates, and GATK tools [20] to recalibrate base quality scores. We then used VarDict [21], SNVer [22], and LoFreq [23] to call variants and Funcotator [20] for annotation. Variants were filtered out if they had fewer than four reads; had a read depth of less than 200 or more than 3000; were not assigned to Chromosomes 1–22, X, or Y; and had a VAF of less than 2%.

### 2.3. DNA Purification for Germline Testing

Germline DNA was isolated from dry frozen skin biopsies using the DNeasy Blood and Tissue Kit (Qiagen #69504), according to the manufacturer′s protocol. Before isolation, any tissue containing blood was removed, and biopsies were washed in PBS (Gibco 10010023).

### 2.4. PCR and Sanger Sequencing

PCR was performed with EconoTaq PLUS GREEN 2X Master Mix (Lucigen #30033‐1) on a Veriti 96‐Well Thermal Cycler (Applied Biosystems) as per the manufacturer′s protocol. PCR conditions were used in initial denaturing for 1 min at 94°C; then 30 cycles of 94°C for 10 s, 60°C–62°C for 30 s, and 72°C for 15 s; and a final post‐PCR extension for 5 min at 72°C. Primers were designed using Primer3 to amplify the desired region of *DDX41*. A list of primer properties is available upon request. Bidirectional Sanger sequencing (Eurofins Genomics) was performed in PCR‐amplified regions of *DDX41*, depending on the location of the variant detected with NGS. Chromatograms were analyzed using the GEAR genome analysis server [24].

### 2.5. Cell Culture

K562 cells were cultured in Roswell Park Memorial Institute 1640 medium (ATCC #30‐2001) supplemented with 10% (*v*/*v*) fetal bovine serum (Thermo Fisher Scientific #12389802) and penicillin–streptomycin (Gibco #15140122).

### 2.6. *DDX41* Monoallelic K562 Cells (K562‐*DDX41* ^+/−^)

To generate DDX41 monoallelic K562 cells, we used a dual‐gRNA‐directed CRISPR‐Cas9 approach to excise an 8852 kb region encompassing the gene. The upstream region of *DDX41* was targeted using a guide (g)RNA system with gRNA‐1, which cuts at chr5:177510525 corresponding to 1266 bp downstream of the coding region of *DDX41*, and with gRNA‐12, which cuts at chr5:177519337 corresponding to 2392 bp upstream of the coding region of *DDX41*. gRNAs were designed for *S. pyogenes* Cas9 with the online software Benchling (https://benchling.com). We chose to edit areas without repeats to avoid off‐target effects and avoided areas outside the exon and promoter regions of *DDX41* to minimize biological effects of editing. The CRISPR‐Cas9 was introduced into the cells via electroporation as a ribonucleoprotein (RNP) complex. For nucleofection of 1 × 10^6^ K562 cells, 250 pmol of each Alt‐R crRNA (Integrated DNA Technologies) (gRNA‐1 and gRNA‐12) and 500 pmol of tracrRNA (Integrated DNA Technologies) were mixed and incubated for 10 min at room temperature to allow complex formation. Next, 153 pmol of Cas9 protein (Integrated DNA Technologies) was mixed with the crRNA:tracrRNA duplexes and incubated for an additional 10 min. Cells were then resuspended in 80 *μ*L of electroporation solution (Cell Line Nucleofector Kit V, Lonza #VCA‐1003) and added to the RNP. Finally, the cell suspension was transferred to a nucleocuvette and electroporated in a Lonza 2B‐Nucleofector device using the T‐003 program. Three days postnucleofection, single cells were sorted into 96‐well plates using a FACS Aria III instrument (BD Biosciences) and expanded to clonal cell lines. Clones were genotyped with PCR using primers amplifying either the *DDX41* deletion junction or Exon 8 or 11 (Table S2). The PCR products were examined by gel electrophoresis and validated with Sanger sequencing (Eurofins Genomics).

### 2.7. CRISPR‐Select

We used a CRISPR‐Select cell‐based variant knock‐in assay to functionally assess the pathogenicity of germline variants. As we have recently reported the principle of the method [16, 17], we will only describe the setup briefly. gRNAs were designed such that the base pairs to be mutated were located as close as possible to the genomic cut site for Cas9 to enhance knock‐in efficiency and located within the protospacer adjacent motif (PAM) or the 1–10 bp seed region of the gRNA target site for the mutations to effectively destroy the Cas9 target site after knock‐in (Table S3). Single‐stranded oligodeoxynucleotide (ssODN) repair templates encoding mutations to be knocked in were designed such that the synonymous WT ^′^ control mutation was placed within the same codon as the variant of interest to promote knock‐in at similar frequencies (Table S4). For WT ^′^, the Human Splicing Finder online tool was used to assess that the mutation did not create a splice site; only mutations with a score below 80 were considered [25]. gRNAs were used in the form of crRNA:tracrRNA duplexes purchased from IDT and reconstituted in nuclease‐free duplex buffer at 100 *μ*M. For RNP generation, Alt‐R *S. pyogenes* Cas9 Nuclease V3 from IDT (#1081059) was used. ssODN repair templates were purchased from IDT as unmodified Ultramer DNA oligonucleotides at 100 *μ*M in IDTE, pH 8.0.

For a nucleofection of 2 × 10^5^ K562 cells, 200 pmol each of crRNA and tracrRNA was mixed and incubated for 10 min at room temperature to allow complex formation. Next, 62 pmol of Cas9 protein was mixed with the crRNA:tracrRNA duplexes and incubated for an additional 10 min. Cells were then resuspended in SF Cell Line Nucleofector (Lonza) Solution and added along with the RNP and 150 pmol of the variant and WT ^′^ ssODN. Finally, the cell suspension was transferred to a 16‐well Nucleocuvette strip (Lonza) and electroporated in a Lonza 4D‐Nucleofector device using the FF‐120 program. All variant assays were performed in triplicate with separate nucleofections, culture wells, and sequencing library preparations.

To enhance knock‐in, we used the DNA‐PK inhibitor, AZD7648, which inhibits nonhomologous end joining repair of CRISPR‐Cas9 DNA cuts and, thereby, favors repair by homology‐directed repair using the ssODNs. An optimal concentration was determined by adding AZD7648 to the culture at concentrations from 0 to 10 *μ*M, 2 h prior to nucleofection. DNA extraction was performed as described below, and PCR and Sanger sequencing were performed as described previously using EconoTaq PLUS GREEN 2X Master Mix (Lucigen #30033‐1). Knock frequency was measured using ICE analysis [26].

Genomic DNA was extracted on Days 2, 14, and 30 after nucleofection from an aliquot of the cell cultures using the Quick‐DNA Microprep kit (Zymo) as per the manufacturer′s protocol. The genomic target site was amplified for NGS analysis using 50 ng of genomic DNA as template and a two‐round PCR protocol consisting of an initial target‐specific amplification step and a separate final PCR adding barcoded sequencing adapters for multiplex sequencing: Primer pairs for amplification of the target site in the first PCR were designed to anneal 40–120 nt outside the region covered by the ssODN repair template and to generate PCR products of 230–350 bps, using Primer‐BLAST3 from NCBI (https://www.ncbi.nlm.nih.gov/tools/primer-blast/). The two‐round PCR was performed as previously described [16, 17]. After pooling roughly equal amounts of second‐round PCR products, the amplicons were sequenced using the MiSeq Reagent Micro Kit v2 300‐cycles (Illumina #MS‐103‐1002) with a 10% spike in PhiX (Illumina #FC‐110‐3002) on a MiSeq instrument (Illumina) with paired‐end 151 bp read configuration. Sequencing depths ranged from 20,000 to 200,000 reads per sample. Demultiplexed NGS data were analyzed by the CRISPResso2 online tool using default settings [27].

### 2.8. Statistics

When comparing patient characteristics, the chi‐square test was used to assess for significant differences in categorical variables. For continuous variables, the Kruskal–Wallis test was applied, as data did not meet the assumptions of normality. In the functional assay, variant/WT ^′^ ratios were compared with a two‐tailed paired *t*‐test. *p* < 0.05 was considered significant. All statistical analyses were performed in R using Version 3.6.1.

## 3. Results

### 3.1. Identifying *DDX41* Variants

**Figure 1:**
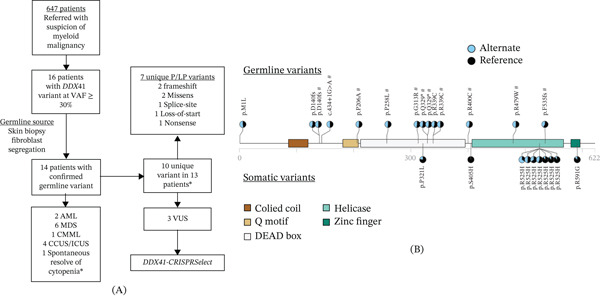
(A) Flowchart of patient cohort and variant curation. ∗One patient had a spontaneous resolution of cytopenia at sampling and was, therefore, excluded from further analysis (B) Lollipop chart of *DDX41* variants identified. Germline variants are on the top, and somatic variants are on the bottom. Variants confirmed by either Sanger sequencing or segregation analysis to be germline are marked with “#.” VUS = variant of uncertain significance; MDS = myelodysplastic syndrome; AML = acute myelogenous leukemia; CCUS = clonal cytopenia of unknown significance; ICUS = idiopathic cytopenia of unknown significance; CMML = chronic myelomonocytic leukemia.

As part of the PTH, we sequenced bone marrow or peripheral blood cell samples from 647 Danish patients, referred to a hematological department with a targeted NGS panel of 145 genes, and found a total of 16 unique *DDX41* variants in 30 patients. In 16 of 30 patients, the *DDX41* variant showed VAF greater than 30%, suggesting a germline origin. We performed Sanger sequencing on DNA derived from skin biopsies and confirmed germline status in nine patients. In addition, one patient had a germline variant confirmed by segregation analysis and another by Sanger sequencing of DNA from skin‐derived fibroblasts obtained in the clinic (Figure 1A). For the remaining patients, we applied the method described by Makishima et al. to predict germline status, identifying three additional patients with likely germline variants. In total, 14 patients were found to carry germline DDX41 variants (Figure 1B), of whom six also harbored a somatic DDX41 variant. In one of the patients, the cytopenia spontaneously resolved; thus, this patient was excluded from further analysis due to being asymptomatic. Across the remaining 13 patients, we identified 10 unique germline variants. The remaining 16 patients were considered to have somatic DDX41 variants and were excluded from subsequent analyses.

### 3.2. Curation of *DDX41* Variants

We classified variants using a pragmatic approach because strict ACMG/AMP classification can be challenging in routine practice, particularly for *DDX41*, where the disease mechanism remains incompletely understood. Variants were deemed LP/P if they fulfilled one of four criteria: (1) classified as LP/P in ClinVar; (2) co‐occurring with a somatic p.R525H variant in another patient in the cohort, without other *DDX41* variants; (3) truncating variants; and (4) reported to have a significantly increased odds ratio (OR) by either Makishima et al. or Kovilakam et al. [2, 3]. Variants were deemed as non‐LP/P if they fulfilled any of three criteria: (1) reported as likely benign/benign in ClinVar, (2) synonymous variants (excluding splice‐site variants), and (3) reported to have an OR close to one by either Makishima et al. or Kovilakam et al. [2, 3]. Seven unique variants in 10 patients were deemed to be LP/P, and four variants in four patients did not fulfill criteria for either LP/P or non‐LP/P variants and were deemed variants of uncertain significance (VUSs). We selected two VUSs for further functional study.

The frequency of LP/P *DDX41* germline variants ranged from 0.4% in patients diagnosed with ICUS to 2.8% in patients diagnosed with MDS (Table 1). When including *DDX41* VUS, the percentage ranged from 0.4% in ICUS to 3.3% in MDS and CCUS. We then compared the clinical characteristics of *DDX41* ^WT^ and *DDX41^g^* patients. Examining the entire cohort, we observed a significantly lower number of leukocytes in patients with a LP/P *DDX41* germline variant (Table 2). Focusing on patients diagnosed with MDS or AML, we found no significant clinical differences, although there was a nonsignificant higher frequency of normal cytogenetic findings in patients with a LP/P *DDX41* germline variant compared to patients without a LP/P *DDX41* variant in line with previous reports (Table 3). The number of patients with *DDX41* variants was too low to compare differences in somatic mutational landscape between *DDX41* ^WT^ and *DDX41^g^* patients. A full characterization of *DDX41^g^* patients can be found in Table S5; furthermore, a full variant classification according to ACMG/AMP guidelines adapted to *DDX41* by Zhou et al. can be found in Table S6[28].

**Table 1:** Incidence of germline *DDX41* variants according to diagnostic subgroups of myeloid cancer.

Diagnosis	*n*, patients with germline P/LP *DDX41* variants	Ratio of patients harboring P/LP *DDX41* variants (%)	*n*, patients with germline *DDX41* variants (e.g., VUS)	Ratio of patients (%) harboring germline *DDX41* variants (e.g., VUS)
MDS	5	2.8	5	3.3
AML	2	2.7	2	2.7
CMML	0	0	1	2.4
CCUS	2	2.2	3	3.3
ICUS	1	0.4	1	0.4

Abbreviations: AML, acute myelogenous leukemia; CCUS, clonal cytopenia of unknown significance; CMML, chronic myelomonocytic leukemia; ICUS, idiopathic cytopenia of unknown significance; MDS, myelodysplastic syndrome.

**Table 2:** Baseline characteristics of all patients with and without a P/LP germline *DDX41*.

	*DDX41* variant		*p*	Test
	Germline variant	WT		
No.	10	639		
Diagnosis (%)			0.341	
MDS	5 (50.0)	175 (27.4)		
AML	2 (20.0)	73 (11.4)		
CCUS	2 (20.0)	89 (13.9)		
CMML	0 (0.0)	41 (6.4)		
ICUS	1 (10.0)	246 (38.6)		
Age (mean [SD])	70.30 (10.77)	68.81 (13.30)	0.725	
Sex = male (%)	7 (70.0)	406 (63.8)	0.943	
Platelet count 10^9^/mL (median [IQR])	127.00 [94.75, 217.25]	121.50 [74.00, 197.00]	0.575	Nonnorm
Hemoglobin (g/dL) (median [IQR])	11.04 [9.91, 12.03]	11.11 [9.35, 13.06]	0.876	Nonnorm
Leucocytes 10^9^/mL (baseline) (median [IQR])	2.60 [1.70, 3.38]	4.50 [2.90, 7.24]	0.016	Nonnorm
LDH (median [IQR])	190.50 [181.25, 203.25]	204.00 [172.00, 250.00]	0.316	Nonnorm
Blast percentage bone marrow (baseline) (median [IQR])	10.50 [1.50, 15.00]	2.00 [0.00, 12.00]	0.387	Nonnorm
Cytogenetic findings (%)			0.199	
No	9 (90.0)	419 (68.7)		
Yes	0 (0.0)	149 (24.4)		
Unknown	1 (10.0)	42 (6.9)		

Abbreviations: AML, acute myelogenous leukemia; CCUS, clonal cytopenia of unknown significance; CMML, chronic myelomonocytic leukemia; ICUS, idiopathic cytopenia of unknown significance; IQR, interquartile range; MDS, myelodysplastic syndrome; SD, standard deviation.

**Table 3:** AML/MDS, baseline characteristics of patients with AML or MDS with and without a P/LP germline *DDX41* variant.

	*DDX41* variant		*p*	Test
	Germline variant	WT		
No.	7	248		
AML (%)	2 (28.6)	73 (29.4)	1	
MDS (%)	5 (71.4)	175 (70.6)	1	
Age (mean (SD))	73.00 (8.08)	70.91 (11.29)	0.579	
Sex = male (%)	4 (57.1)	156 (63.2)	1	
Platelet count 10^9^/mL (median [IQR])	119.00 [98.50, 201.50]	116.00 [61.00, 196.00]	0.494	Nonnorm
Hemoglobin (g/dL) (median [IQR])	11.44 [10.71, 12.25]	9.67 [8.49, 11.12]	0.028	Nonnorm
Leucocytes 10^9^/mL (baseline) (median [IQR])	2.70 [1.60, 5.35]	4.25 [2.47, 7.03]	0.189	Nonnorm
Blast percentage bone marrow (baseline) (median [IQR])	12.50 [9.75, 19.00]	4.00 [1.00, 25.00]	0.236	Nonnorm
Cytogenetic findings (%)			0.065	
No	7 (100.0)	134 (55.6)		
Yes	0 (0.0)	98 (40.7)		
Unknown	0 (0.0)	9 (3.7)		

Abbreviations: AML, acute myelogenous leukemia; IQR, interquartile range; MDS, myelodysplastic syndrome; SD, standard deviation.

### 3.3. Generation of *DDX41* Monoallelic K562 Assay Cell Line (K562‐*DDX41* ^+/−^)

**Figure 2:**
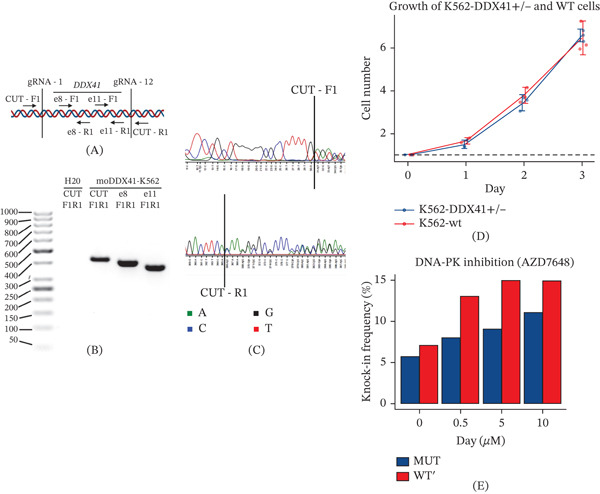
Creation of a *DDX41* monoallelic K562 clonal cell line (K562‐*DDX41* ^+/−^). (A) Schematic representation of gRNA and primer placement. (B) PCR of K562‐*DDX41* ^+/−^ to confirm editing; H_2_O was used as a negative control. (C) Sanger sequencing of the CUT PCR product showing a frameshift near the cut site, confirming more than one edit with different repair outcomes. (D) Growth comparison of WT K562 and K562‐*DDX41* ^+/−^. (*n* = 3). (E) Titration of the DNA‐PK inhibitor AZD7648 with simultaneous knock‐in of a variant and a synonymous control (*n* = 1 for each dose).

To develop a functional assay for *DDX41* variants, we first searched the literature to identify a cell line that expresses and is dependent on DDX41 for proliferation. Shinriki et al. showed that DDX41 is expressed in the K562 lymphoblast cell line, and when knocked down with *DDX41*‐shRNA, proliferation decreased [12]. This reduced proliferation was attributed to impaired DNA replication and delayed G2/M transition, leading to genomic instability and mitotic abnormalities in DDX41‐deficient cells [12]. K562 cells have three wild‐type (WT) copies of *DDX41* [29], and to make a more robust *DDX41* variant assay, we used CRISPR‐Cas9 targeted knockout to generate a monoallelic *DDX41* K562 clone cell line, termed K562‐*DDX41* ^+/−^. Specifically, cells were transfected with a dual gRNA system targeting the upstream and downstream region, gRNA‐1, see Figure 2A. We chose to cut areas without repeats to avoid off‐target effects and avoided areas outside the exon and promoter regions of *DDX41* to minimize biological effects of editing, such as expression. Cells were transfected with both guides simultaneously using electroporation, seeded into 96‐well plates as singlets, and then expanded to clonal cell lines. We screened the clones for deletion of *DDX41* excision events with concomitant preservation of a WT on the remaining allele, using a PCR and gel electrophoresis approach (Figure 2B). The cut products of select clones were then Sanger‐sequenced to identify cases where two alleles were edited with two slightly different repair outcomes, as indicated by out‐of‐phase chromatogram traces (Figure 2C). There was no difference in proliferative capacity between K562‐*DDX41* ^+/−^ and unedited K562 cells (Figure 2D).

### 3.4. *DDX41*‐CRISPR‐Select: Functional Impact of Individual *DDX41* Variants

**Figure 3:**
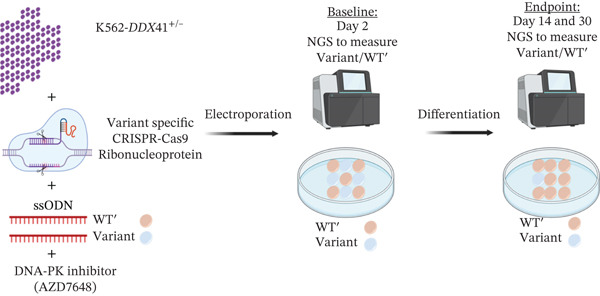
*DDX41*‐CRISPR‐Select assay setup. ssODN = single‐stranded oligodeoxyribonucleotide.

**Figure 4:**
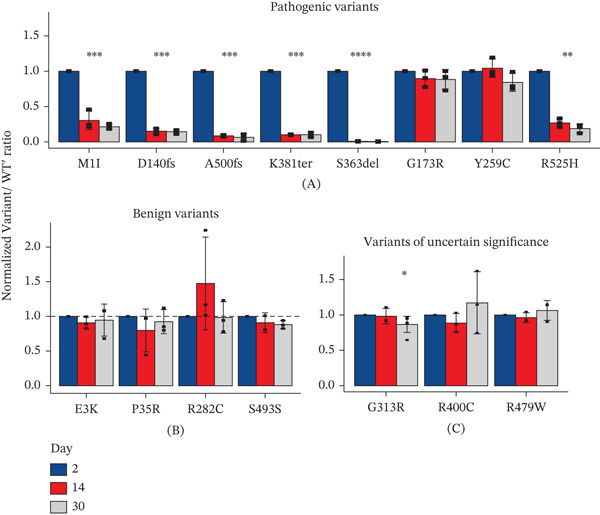
Results from *DDX41*‐CRISPR‐Select. All variants were tested in triplicate. The *y*‐axis shows the ratio of read counts between the variant of interest and the WT ^′^ (synonymous variant), used to estimate the effect on proliferation. Ratios were normalized to the mean ratio observed on Day 2. (A) Results for known pathogenic variants. (B) Results for known benign variants. (C) Results for variants of uncertain significance identified in our patient cohort. G313R displayed a marked decrease in read counts between Days 2 and 30, but not between Days 2 and 14. ^∗^ *p* < 0.05,  ^∗∗^ *p* < 0.01,  ^∗∗∗^ *p* < 0.001, and  ^∗∗∗∗^ *p* < 0.0001.

To establish a CRISPR‐Select functional assay for *DDX41* variants, we used the K562‐*DDX41* ^+/−^ cell line with cell proliferation and survival as assay readout. Proliferation and survival were chosen as readout, because they can be accurately quantified by a CRISPR‐Select assay, due to the previously described findings by Shinriki et al. To evaluate specific variants, we electroporated K562‐*DDX41* ^+/−^ cells with a CRISPR‐Cas9 RNP targeting the genomic site of the variant of interest, along with two ssODN repair templates coding either for the variant or a neutral synonymous control in the same codon, termed WT ^′^. We first optimized for high knock‐in frequencies to ensure a quantitative assay with a large dynamic window using the DNA‐PK inhibitor AZD7648 (Figure 2E). The result of CRISPR‐Select editing was a heterogeneous population of cells containing both variant cells and WT ^′^ cells growing in the same culture (Figure 3). We then measured how each variant affected proliferation compared to the neutral control. After DNA was extracted from an aliquot of cells, we performed genomic PCR amplification of the edited target site followed by targeted NGS to measure the absolute frequencies of variant and WT ^′^ cells in the cell culture. We used the frequencies on Day 2 as a baseline measure and compared this to Days 14 and 30. Based on previous reports that *DDX41* knockdown in K562 significantly decreases proliferation, we expected the frequency of pathogenic variants to decrease relative to their respective neutral controls over time. We selected pathogenic variants spanning the entire *DDX41* gene and representing different types of variants. All selected pathogenic variants, except for S363del, are classified as LP/P in ClinVar. S363del was included as a pathogenic variant due to an observed high OR reported by Makishima et al. [2]. As expected, all tested pathogenic variants impaired proliferation, as compared to the neutral control (Figure 4A). Truncating variants, M1I, D140fs, A500fs, and K381ter, and the in‐frame deletion, S363del, showed 75%–99% decrease in the normalized variant/WT ^′^ ratio from baseline to Day 30. The somatic variant, R525H, exhibited a 57% decrease, while the missense pathogenic germline variants, G173R and Y259C, showed a decrease of 12% and 16%, respectively. The change over time was statistically significant for the truncating variants, the in‐frame deletion, and the R525H variant (all *p* *v* *a* *l* *u* *e* *s* < 0.01), but not for G173R or Y259C. We then tested how benign variants performed in the assay. As only synonymous variants were classified as likely benign/benign in ClinVar at the time of the design of the experiment, we chose missense or synonymous variants reported to have OR near 1 as reported by either Makishima et al. [2] or Kovilakam et al. [3]. As expected, none of these variants, which are located at different regions of *DDX41*, impaired proliferation compared to the neutral control (Figure 4B). Finally, we examined the three germline VUSs, G313R, R400C, and R479W, found in the Danish cohort, to assess pathogenicity. Whereas R400C and R479W did not affect proliferation, G313R caused a significant 16.7% decrease in proliferation comparable to the two pathogenic missense variants, G173R and Y259C (Figure 4C). Cells with frameshift InDels in *DDX41*, which are introduced via nonhomologous end joining or microhomology‐mediated repair pathways, served as an internal positive control in our *DDX41* CRISPR‐Select assay, where their negative selection over time—except for M1I, where InDels were introduced outside the reading frame—validated the functional neutrality of the tested apparently benign variants, when compared to the WT ^′^ control (Figure S1). The internal positive assay control for the P35R edit site showed a nonsignificant decrease in frameshift InDels over time, suggesting limited negative selection and indicating that the results of the assay for P35R should be interpreted with caution.

## 4. Discussion

In this study, we were able to identify a LP/P *DDX41* germline variant in 2.7% of patients diagnosed with MDS/AML. This frequency is comparable to a previous European study, which identified LP/P *DDX41* germline variants in 2.4% of patients with either MDS or AML [2, 5]. Furthermore, we investigated the frequency of LP/P germline *DDX41* variants in CCUS and found a similar frequency of 2.2%, indicating that *DDX41* germline variants are implicated in a substantial number of patients with CCUS. This is supported by multiple reports of cytopenia preceding malignancy in patients with *DDX41* germline variants. Some of the variants detected in this Danish cohort were similar to previously reported variants from cohorts of European patients (e.g., p.M1I and p.D140fs), and we did not detect variants previously reported as more frequent in cohorts of patients from Asia (e.g., the p.A500fs variant) [2]. *DDX41* variants were almost absent in patients diagnosed with ICUS.

We chose a pragmatic approach for variant curation and incorporated both curated databases, such as ClinVar, and previously reported OR from the two large studies by Makishima et al. and Kovilakam et al. This only helped us curate half of the missense variants, reflecting that many *DDX41* variants are unique, making curation and genetic counseling challenging in a clinical setting. Therefore, an accurate method to predict the functional effect of variants is needed, and in this study, we pursued an experimental approach with CRISPR‐Select [16, 17]. CRISPR‐Select is a powerful way to investigate the pathogenic potential of individual variants [17]. Experimental artifacts and CRISPR off‐target action are minimized by using an internal neutral control in the assay, and the positive frameshift InDel control can validate the neutrality of apparently benign variants. Variants are being expressed by cells in a relevant genomic context instead of overexpression by a vector, which can hide the effect of missense variants, as previously shown by Funk et al. for *TP53* missense variants [30]. Lastly, results are based on hundreds or thousands of different knock‐in events, as editing is culture‐based instead of establishing individual clones with the risk of unknown biases. We report the first demonstration of diminished proliferation and survival when examining pathogenic variants by *DDX41* CRISPR‐Select and no effect of benign *DDX41* variants by employing the first functional assay for *DDX41*. Interestingly, there seems to be a clear difference in the proliferative impairment caused by truncating compared to missense variants, with the former class showing the strongest effect, although the somatic hotspot missense variant p.R525H showed a similar profound effect on proliferation and survival. Clinical observations also support a difference between truncating and missense variants, as Makishima et al. found a higher incidence of progression to AML in patients harboring truncating variants [2] and Kovilakam et al. found a higher OR for MDS and AML for truncating compared to missense variants [3]. Given the limited understanding of the DDX41 protein′s function and its role in disease, it is plausible that certain missense variants may exert pathogenic effects through mechanisms not detectable by our current assay readout, and additional research is needed to unravel the biological mechanism of action of missense variants. Lastly, we found that one of the VUSs, p.G313R, had a significant effect on proliferation and survival. This variant was found in a 68‐year‐old male patient with low‐risk MDS with pancytopenia and no family history of MN. The variant was detected in gnomAD at a variant allele frequency of 0.00034%. Furthermore, Kovilakam et al. identified one healthy carrier in the UK Biobank. They did, however, also find that 1/18 patients carrying the p.G313S variant developed MN with a co‐occurring somatic *DDX41* variant [3]. Taken together, these data suggest that the p.G313R variant is pathogenic in line with the results from our assay.

Well‐validated functional assays can provide strong evidence toward pathogenic or benign classification of a VUS when formal statistical analysis permits calculation of a likelihood ratio toward pathogenicity [31, 32]. Expanding the set of control variants (at least 11 pathogenic and benign variants according to Bose et al. [32]) and applying such rigorous statistical analysis could readily extend this proof‐of‐concept assay to a well‐established clinical variant classification tool for testing additional VUSs, as was done for BRCA2 variants using CRISPR‐Select^33^.

Our *DDX41* CRISPR‐Select assay can easily be modified to evaluate how *DDX41* variants affect other cellular processes than proliferation and survival by combining the assay with FACS using a marker for a cellular process of interest and comparing variant/WT ^′^ ratios in cell populations with low and high marker levels, as previously shown [17]. For instance, the assay can test the effect of variants on the ability of DDX41 to resolve R‐loops by combining CRISPR‐Select with FACS using an antibody directed against R‐loops [17]. Furthermore, by performing our assay in the absence or presence of a chemical compounds in the culture, it can also readily be used to measure the effect of novel targeted treatments (e.g., testing compounds specific for cells with p.R525H), which was proposed as a possible treatment strategy by Winston et al. [10]. Finally, CRISPR‐Select could serve as a clinical assay to assess the functional impact of individual germline variants in a patient‐specific context, enabling personalized insights from patient to patient.

In conclusion, we generated a monoallelic *DDX41* K562 cell line and developed a CRISPR‐Select assay to functionally assess *DDX41* variants. While the assay revealed clear differences primarily driven by truncating pathogenic variants, missense germline variants showed more subtle effects on proliferation and survival, consistent with hypomorphic phenotypes. Additionally, we identified one *DDX41* VUS associated with a significant reduction in proliferation and survival, suggesting potential pathogenicity, whereas two other VUSs showed no measurable impact, supporting a likely benign classification.

## Author Contributions

N.J.N., M.A., C.C.L., J.S.J., M.F., J.W., B.P., and K.G. conceived and designed the experiments. N.N., M.A., I.I.I., and J.S.J. performed the experiments. N.N., M.A., M.F., M.K.A., and K.G. interpreted the data. J.W.H., K.R.‐J., C.S., M.T.S., A.S.R., and C.A.S. handled patient material and collected clinical data. N.N., M.K.A., and K.G. wrote the manuscript. All authors critically reviewed the final version of the manuscript.

## Funding

This study was funded by the Rigshospitalet′s Research Foundation (to N.N.) and the Danish Cancer Society, R302‐A17259. Further funding came from a center grant from the Novo Nordisk Foundation (Novo Nordisk Foundation Center for Stem Cell Biology, Dan Stem; NNF17CC0027852) and the Greater Copenhagen Health Science Partners (Clinical Academic Group in Translational Hematology). The project is part of the Danish Research Center for Precision Medicine in Blood Cancers, which is funded by the Danish Cancer Society, R223‐A13071. M.F. received funding from the Danish Childhood Cancer Foundation (Grant No. 2023‐001159, 2024‐001324, and 2025‐001472).

## Ethics Statement

An ethics statement and a description of the patient consent procedure can be found in the first paragraph of the Methods section.

## Conflicts of Interest

University of Copenhagen submitted on December 4, 2020, a patent application (EP20211801.4) with inventor M.F. for CRISPR‐Select. M.F. is the cofounder of Biophenyx that uses CRISPR‐Select assays. Claudia Schöllkopf serves on the advisory board for Incyte Biosciences Distribution and has received travel grants from Northon Healthcare Limited, Swedish Orphan Biovitrum, and AbbVie.

## Supporting Information

Additional supporting information can be found online in the Supporting Information section.

## Supporting information


**Supporting Information 1.** Figure S1: Assay control from *DDX41*‐CRISPR‐Select. For each variant, we also introduce Frameshift InDels due to nonhomologous end joining repair mechanisms. We expect the frequency of frameshift variants to decrease over time compared to SYN, except for the variant M1I, as frameshift variants in this site are outside the coding sequence. Negative selection for Frameshift InDels in the dish of neutral variants validates that those variants are indeed functionally normal. All variants were tested in triplicate. (A) Control for pathogenic variants. (B) Control for known benign variants. (C) Control for variants of uncertain significance identified in our patient cohort.  ^∗^ *p* < 0.05,  ^∗∗^ *p* < 0.01,  ^∗∗∗^ *p* < 0.001, and  ^∗∗∗∗^ *p* < 0.0001.

**Supporting Information 2.** Table S1: Overview of genes included in the targeted sequencing panel. This includes gene symbols, Ensembl stable IDs, NCBI gene IDs, and chromosomal locations. Each gene is annotated for inclusion in a curated list and target region used for sequencing (coding region only, i.e., no flanking base pairs). Table S2: Primer sequences used for DDX41 CRISPR‐Select editing. This includes primer names and sequences (5 ^′^–3 ^′^). Adapter sequences for two‐step PCR before NGS (next‐generation sequencing) are italicized. Table S3: List of guide RNAs (crRNAs = CRISPR RNAs) used for DDX41 CRISPR‐Select editing. This includes the guide RNA name and crRNA sequence (5 ^′^–3 ^′^). Table S4: List of single‐stranded oligodeoxynucleotides (ssODNs) used for DDX41 CRISPR‐Select. This includes reference and mutant ssODNs and their full sequences. Table S5: Clinical characteristics of patients with germline DDX41 variants. Data includes age, sex, diagnosis, family history, karyotype, germline variant, variant curation (P/LP = pathogenic/likely pathogenic; VUS = variant of uncertain significance), curation criteria (e.g., ClinVar), somatic variants, blast count in bone marrow (%), leukocytes (10^9^/L), neutrophil granulocytes (10^9^/L), hemoglobin (g/dL), and platelet count (10^9^/L). Table S6: Variant curation, according to ACMG/AMP guidelines, adopted to DDX41 by Matusi et al. (PMID: 38492200).

## Data Availability

It is not possible to deposit the data in public repositories, since these data are considered sensitive personal data according to Danish law and the European Union General Data Protection Regulation (GDPR), and thus cannot be shared with third parties without prior approval. To access the data, please contact the corresponding author at kirsten.groenbaek@regionh.dk. Access can only be granted for research purposes and only if a data processor or data transfer agreement can be made in accordance with Danish and European law at the given time. The expected timeframe from response until access is granted is ~6 months.
