# Supplementary material for: Exploring the Functional Impact of Individual DDX41 Variants With a Fast and Robust Cell‐Based Method
Source: Hum Mutat. 2026 Jun 25;2026:3758915. doi: 10.1155/humu/3758915 (PMC13303339; doi:10.1155/humu/3758915)
Supplement: Supplementary file 1 — Supporting Information 1. Figure S1: Assay control from DDX41‐CRISPR‐Select. For each variant, we also introduce Frameshift InDels due to nonhomologous end joining repair mechanisms. We expect the frequency of frameshift variants to decrease over time compared to SYN, except for the variant M1I, as frameshift variants in this site are outside the coding sequence. Negative selection for Frameshift InDels in the dish of neutral variants validates that those variants are indeed functionally normal. All variants were tested in triplicate. (A) Control for pathogenic variants. (B) Control for known benign variants. (C) Control for variants of uncertain significance identified in our patient cohort. ∗ p < 0.05, ∗∗ p < 0.01, ∗∗∗ p < 0.001, and ∗∗∗∗ p < 0.0001. [file HUMU-2026-3758915-s002.pptx]

## Slide 1
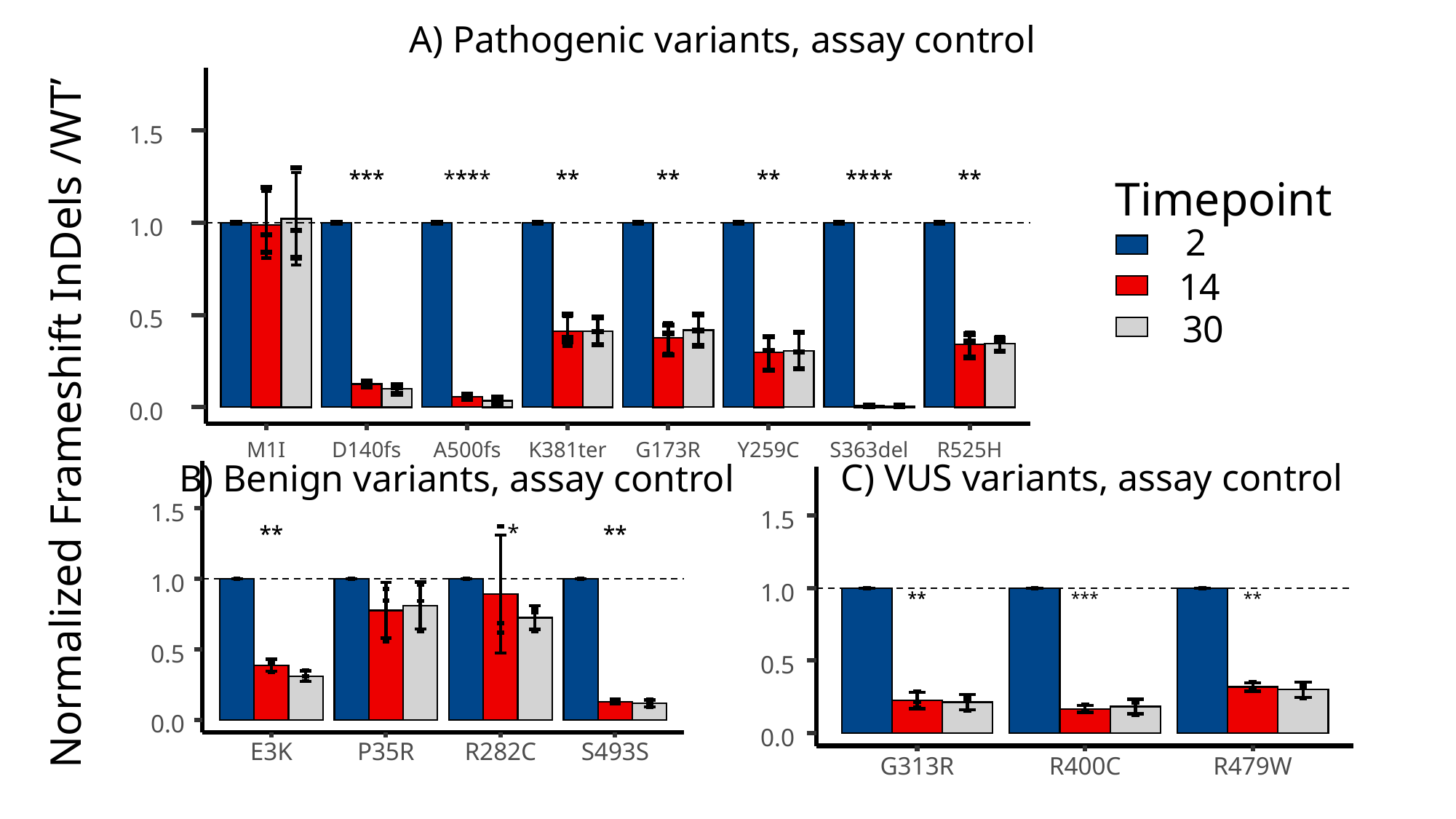

A) Pathogenic variants, assay control
1.5
***
***
****
**
**
**
**
**
**
**
****
****
**
**
Timepoint
1.0
2
14
0.5
30
0.0
G173R
S363del
D140fs
A500fs
Y259C
K381ter
R525H
M1I
Normalized Frameshift InDels /WT’
C) VUS variants, assay control
1.5
1.0
**
**
***
**
0.5
0.0
G313R
R400C
R479W
B) Benign variants, assay control
1.5
*
**
**
**
**
1.0
0.5
0.0
S493S
R282C
E3K
P35R
